# Data resource profile: the Korean Community Health Status Indicators (K-CHSI) database

**DOI:** 10.4178/epih.e2023016

**Published:** 2023-02-02

**Authors:** Hye-Eun Lee, Yeon-gyeong Kim, Jin-Young Jeong, Dong-Hyun Kim

**Affiliations:** 1 Department of Social and Preventive Medicine, Hallym University College of Medicine, Chuncheon, Korea; 2 Institute of Social Medicine, Hallym University College of Medicine, Chuncheon, Korea; 3 Graduate School of Public Health, Seoul National University, Seoul, Korea; 4 Hallym Research Institute of Clinical Epidemiology, Hallym University College of Medicine, Chuncheon, Korea

**Keywords:** Population health, Public health surveillance, Social environment, Health equity

## Abstract

Korean Community Health Status Indicators (K-CHSI) is a model-based database containing annual data on health outcomes and determinants at the municipal level (*si/gun/gu*-level regions, including mid-sized cities, counties, and districts). K-CHSI’s health outcomes include overall mortality, disease incidence, prevalence rates, and self-reported health. Health determinants were measured in 5 domains: socio-demographic factors, health behaviors, social environment, physical environment, and the healthcare system. The data sources are 71 public databases, including Causes of Death Statistics, Cancer Registration Statistics, Community Health Survey, Population Census, and Census on Establishments and Statistics of Urban Plans. This dataset covers Korea’s 17 metropolitan cities and provinces, with data from approximately 250 municipal regions (*si/gun/gu*). The current version of the database (DB version 1.3) was built using 12 years of data from 2008 to 2019. All data included in K-CHSI may be downloaded via the Korea Community Health Survey site, with no login requirement (https://chs.kdca.go.kr/chs/recsRoom/dataBaseMain.do). K-CHSI covers extensive health outcomes and health determinants at the municipal level over a period of more than 10 years, which enables ecological and time-series analyses of the relationships among various health outcomes and related factors.

## INTRODUCTION

Population health is defined as “the health outcomes of a group of individuals, including the distribution of such outcomes within the group” by Kindig & Stoddart [1]. It has also been suggested that population health includes health outcomes, patterns of health determinants, and the policies and interventions that link them. With this perspective, modern public health policies have evolved from focusing on individual risk factors to concentrating on determinants of population health and empowering community participation in promoting population health simultaneously [2]. In the individual risk-factor approach, a population’s health risk is reduced to the sum of the health problems of individuals within the group, and direct biological causes of disease, which are separate from a health problem’s social context, become the most typical point for intervention. In contrast, researchers who support the population-health perspective assert that causes of diseases and health should be determined by comparing the health gap between populations, and that the health and disease patterns of a group’s members may be determined by their social relations [3]. Medical interventions and individual behavioral approaches are inadequate for addressing health inequities [4]. On the contrary, with increasing evidence that social, economic, and environmental determinants of health may impact health outcomes, strategies targeting social determinants are likely to reduce health inequities [5]. This population-health perspective is based on the recognition that historical, cultural, and socioeconomic contexts should be considered to understand how and why individuals in groups are exposed to risky behaviors and become unhealthy [6,7]. From this perspective, when a population group becomes a unit of interest, health interventions may also be made by paying attention to the group’s characteristics, which has significant implications for health promotion strategies and the allocation of healthcare resources [8].

Hence, the target of the intervention shifts from individuals or groups of individuals to their environments—the “settings of everyday life” [2]. These environments and settings refer to the communities in which people live and work.

Because this ecological orientation has been widely adopted, researchers have become interested in studying how health determinants affect community health and why there is a gap in the health of various communities. Additionally, local health departments need health-related data from their own regions to better understand the burden and distribution of health and to plan evidence-based health interventions.

In the United States, to address this demand, large and well-funded population health data projects have been introduced. These include County Health Rankings & Roadmaps (CHR&R) and PLACES (Population Level Analysis and Community Estimates). CHR&R has provided annual county health rankings, or “population health checkups,” for more than 3,000 counties across the United States since 2010 [9]. The rankings are based on a conceptual model of population health comprising health outcomes such as mortality and morbidity, as well as health determinants including health behaviors, clinical care, social and economic factors, and the physical environment. The composite measure scores are calculated using more than 30 weighted indicators at the county level, after which counties are ranked by ordering the composite scores [9].

PLACES is an expansion of the original 500 Cities Project that began in 2015, which provides model-based estimates of 29 measures for all 3,142 counties, 28,484 incorporated and census-designated places, 72,337 census tracts, and 32,409 ZIP code tabulation areas across the United States [10]. Health measures include health behaviors, health outcomes, health status, and prevention practices. The website offers visualizations of geographical health estimates, comparisons between places, and downloadable data for further analysis.

The Community Health Status Indicators Project (CHSI) is a third example of a big-data United States population health project. Since 2000, this tool has provided more than 300 county-level health profiles, including items related to chronic and infectious diseases, birth outcomes, mortality, environmental health, health services, health behaviors, health-related quality of life, vulnerable populations, and health disparities [11]. The project also enables users to compare a county’s health with “peer counties,” a feature introduced in 2008. Although CHSI has produced standardized local health data in easy-to-understand formats for nearly 2 decades, the project was discontinued in 2017, for which many researchers expressed regret [12].

In 2008, the Korea Disease Control and Prevention Agency (KDCA) initiated the Korea Community Health Survey (KCHS), a community-based, cross-sectional survey that provides health-related data by municipal region (*si/gun/gu*, including mid-sized cities, counties, and districts), at the jurisdiction level of a community health center [13]. KCHS has become a useful source of municipal-level health data that were previously unavailable. However, KCHS itself provides health behavior-focused data only through individual questionnaires. As more diverse factors in various categories affect the population’s health level, demand has grown for the establishment of a comprehensive database linking these indicators and reflecting community health and determinants.

Considering this demand, we have constructed a database named Korean Community Health Status Indicators (K-CHSI), which contains annual values at the municipal level.

## DATA RESOURCE

### Community health model

In an approach for causal inferences about health disparity factors between populations, it is important to develop a model based on relationships between population health and health determinants, including social structures and contexts. We reviewed the community health model, which has been theoretically and empirically proven in other institutes, and proposed health determinants of community health from a population-health perspective.

In Dever’s health model reflecting environmental factors, community health was measured by evaluating detailed items from community health status data, health system data, lifestyle data, human biology data, environmental data, socioeconomic indicators, and overall quality of life, in addition to traditional disease and medical service-oriented data [14].

Anderson et al. [15] recognized societal resources (human, social, and financial) and the physical environment (natural resources) as determinants of community health with intermediate outcomes, including neighborhood living conditions, opportunities for learning and developing capabilities, community development and employment opportunities, prevailing community norms, customs and procedures, social cohesion, civic engagement and collective efficacy, health promotion, disease and injury prevention, and healthcare. In this process, equity and social justice in a society affect the pathway by which societal resources and physical environments influence health outcomes, and community health is ultimately determined by the interactions among all these factors [15].

**Figure 1.**
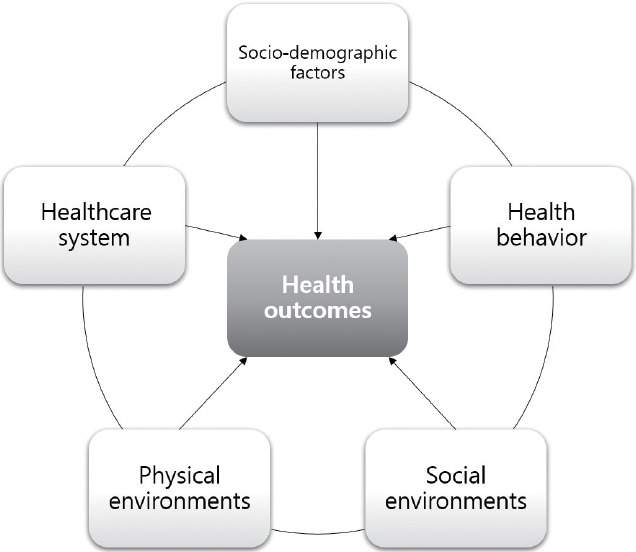
Community health model.

Based on previously proposed models [14-16], health outcomes in K-CHSI were measured by overall mortality, selected disease incidence, prevalence rate, and self-reported health. Additionally, health determinants were measured through 5 domains: sociodemographic factors, health behaviors, social environment, physical environment, and the healthcare system [17]. The proposed community health model in the context of Korea is shown in Figure 1.

### Structure of the database

K-CHSI was established using various open resources for public data. The data sources are 71 public databases, including Causes of Death Statistics, Cancer Registration Statistics, KCHS, Population Census, and Census on Establishments and Statistics of Urban Plans. Selected sources of statistics are listed in Tables 1 and 2, and a full list can be found in the database description on the KDCA website, where the database may be downloaded. This dataset covers Korea’s 17 metropolitan cities and provinces, with data from approximately 250 municipal regions (*si/gun/gu*). The dataset includes estimates for 2,370 measures: 1,147 health outcomes, 402 health behaviors, 497 measures relevant for healthcare systems, 93 socio-demographic factors, 149 aspects of the physical environment, and 83 aspects of the social environment. The current version of the database (DB version 1.3) is built using 12 years of data from 2008 to 2019, although the variables collected vary slightly by year and data source.

### Composition of the database

**Figure 2.**
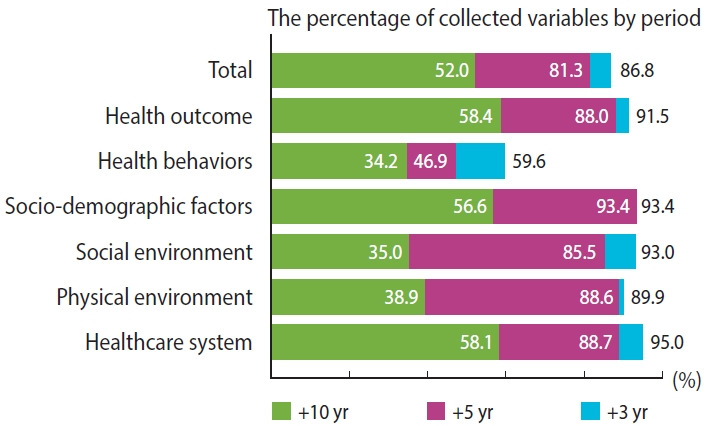
Proportions of data according to the period of coverage.

The database comprises municipal-level health outcomes and determinant variables. Health outcome data were classified into 4 categories: mortality rate, incidence rate, prevalence, and self-reported health status (Table 1). Health determinant variables were categorized into 5 areas: socio-demographic factors, health behaviors, social environment, physical environment, and the healthcare system (Table 2). The first version of the database included well-established health determinants according to a thorough literature review [17]. For subsequent updates, researchers proposed many related indicators, whose relevance for addition is evaluated annually. The data quality control committee composed of public health experts was established by the KDCA. Detailed variables by category are presented in Supplementary Materials 1 and 2. The database covers the years 2008-2019, and approximately 60% of the variables have observations from more than 10 years (Figure 2).

## DATA RESOURCE USE

Previous studies on community-level health have mainly used KCHS data. A study on factors related to regional variation in high-risk drinking rates found that the current smoking rate, perceived stress rate, crude divorce rate, and financial independence rate of all municipalities were related to the high-risk drinking rate [18]. Community-level data may be used to evaluate the effects of specific health policies. For example, a study used municipal-level data from the KCHS to evaluate the effects of community-level smoke-free ordinances on smoking rates in men [19]. It is also common to use data from 2 or more sources for analyzing population-level health. For example, ecological studies on the geographical distribution of liver cancer, gall bladder cancer, and thyroid cancer have used municipal-level data derived from the Cancer Incidence Registry and the KCHS [20,21]. A previous study investigated the influence of area-level factors on adolescent suicide using suicide rates from Korean mortality data and arealevel socioeconomic variables from the Population Census and the Korean Labor & Income Panel Study [22]. This study found that economic status, social fragmentation, and community health services were associated with adolescent suicide rates. K-CHSI covers extensive health outcomes and health determinants at the municipal level over a period of more than 10 years, which enables ecological and time-series analyses of the relationships among various health outcomes and related factors. Additionally, the data may be used for multi-level analysis if combined with individual-level data from another source with data on the same region. Several examples of research topics that may be investigated using this database are presented in Table 3.

The current K-CHSI is built as a database listing various outcome variables and health determinants related to community health levels. In the future, its use should extend to the planning and evaluation of regional community health programs based on scientific evidence for a district’s major health problems. Therefore, K-CHSI needs to be rebuilt as a relational database capable of retrieving local health indicators more quickly and enabling exploratory data analysis by a user-friendly web interface. In addition, the database system should suggest inter-connected indicators related to the input, process, and output of each public health intervention. It should be developed as a problem-solving resource that presents indicators for evaluating specific interventions’ effectiveness.

## STRENGTHS AND WEAKNESSES

K-CHSI has the following strengths. First, its items were carefully selected based on the community-health model, and the validity, reliability, sensitivity, and robustness of the core measures were evaluated when the data were first established. Second, data quality is guaranteed because the independent quality-control committee has evaluated the reliability and appropriateness of the items annually. Third, users can save time and effort in data searching, refining, and processing, since the data have been unified from 71 different sources of public data. Fourth, it is easy to access the data by simply downloading files, without having to apply for data access.

One of K-CHSI’s main weaknesses is variability in item completeness. Not all the variables cover the entire period, and some variables provide observations only for the metropolitan city and province level (*si/do*) without municipal-level details (*si/gun/gu*). Another weakness is that researchers may have difficulty in further processing the data for custom analysis because K-CHSI offers values calculated according to predefined definitions from the original data sources.

## DATA ACCESSIBILITY

All K-CHSI data may be downloaded from the KCHS site (https://chs.kdca.go.kr/chs/recsRoom/dataBaseMain.do). The entire data file may be downloaded without logging in by choosing the button labeled “파일다운로드” (i.e., “File download”). Data files are provided in Excel. There are a total of 4 files, from version 1.0 to 1.3. In addition, the website provides a search system that allows users to select a region and year for a variable of interest, then save their search results.

### Ethics statement

The study was exempt from institutional review board approval because only public open data were used in this study.

## Figures and Tables

**Table 1.** Health outcome variables

Category	Contents (selected)	No. of indicators	Data sources (selected)
Mortality rate	Mortality by cause of death	530	Causes of Death Statistics
Incidence rate	Cancer incidence, infectious disease, injury incidence	400	Cancer Registration Statistics, Notifiable Infectious Diseases Report
Prevalence	Disease experience rate, obesity rate, oral health	63	KCHS, Korea Youth Risk Behavior Survey
Self-reported health	Self-rated health, quality of life, activity limitation	48	KCHS, Korea Youth Risk Behavior Survey
	Stress, depressive symptoms, suicidal thoughts, cognitive function		
Others	Traffic accidents, oral health examination results, violence	106	Traffic Accidents Statistics, Korean Children’s Oral Health Survey, KCHS, Report on Child Abuse

KCHS, Korea Community Health Survey.

**Table 2.** Health determinant variables

Main category	Subcategory	No. of indicators	Data sources (selected)
Socio-demographic factors	Population and household	76	Population Statistics Based on Resident Registration
Health behaviors	Nutrition and diet	41	KCHS, Korea Youth Risk Behavior Survey
	Alcohol	30	
	Health education and public relations	29	
	Personal hygiene	14	
	Mental health related behavior	12	
	Oral hygiene	34	KCHS, Korean Children’s Oral Health Survey
	Physical activity	21	KCHS, Korea Youth Risk Behavior Survey
	Safety awareness	29	KCHS, Korea Youth Risk Behavior Survey, Transport Culture Index
	Smoking	46	KCHS, Korea Youth Risk Behavior Survey
	Screening and vaccination	145	KCHS, Korea Children’s Vaccination Survey
Social environment	Education	10	Statistics of Education
	Economy	26	Census on Establishments, Land Price Statistics
	Culture	3	The Statistics on Environments and Policies of Regional Culture
	Welfare	7	Census on Establishments, Local Finance Report
	Social capital	12	KCHS
	Political participation	3	Election Statistics
	Safety	39	National Police Agency Crime Statistics, Transport Culture Index
Physical environment	Natural environment	42	KCHS, Status and trends of air quality
	Living environment	88	Housing Census, Statistics of Urban Plan
	Working environment	19	Toxics Release Inventory
Healthcare system	Health and medical resources	155	National Health Insurance Statistical Yearbook
	Health care use	308	Medical Use Statistics by Region, Emergency Medical Statistics
	Public health service	34	Basic statistics of local governments

KCHS, Korea Community Health Survey.

**Table 3.** Examples of research topics using K-CHSI

Determinant categories	Examples of research topics
Socio-demographic factors	Association between the proportion of one-person households and the prevalence of depression in communities
	Association between population density and mortality from infectious diseases at the municipal level
Health behaviors	Association between high-risk drinking rates in local communities and traffic accident rates in the population
	The relationship between the rate of community hand-washing practices and the incidence of infectious diseases
Social environment	Effect of the number of pubs per 1,000 residents on the change in community crime rates
	The impact of local community social welfare budgets on quality of life
	Basic pension benefit rates in communities and elderly suicide mortality rates
Physical environment	The association between green area per capita and the suicide rate in communities
	Relationship between the proportion of pedestrian-only roads and obesity rates in local communities
Healthcare system	Impact of the number of medical institutions on the mortality rate of the three major cancers (liver, stomach, and lung cancer) in communities
	Relationship between changes in cancer screening participation rates and changes in selected cancer mortality at the community level

K-CHSI, Korea Community Health Status Indicators.

## SUPPLEMENTARY MATERIALS

Supplementary Material 1. List of health outcome variables

Supplementary Material 2. List of health determinants variables
