# Supplementary material for: Data resource profile: the Korean Community Health Status Indicators (K-CHSI) database
Source: Epidemiol Health. 2023 Feb 2;45:e2023016. doi: 10.4178/epih.e2023016 (PMC10581888; doi:10.4178/epih.e2023016)
Supplement: Supplementary Material 1. — List of health outcome variables [file epih-45-e2023016-Supplementary-1.docx]

**[Supplement 1]**

**Supplementary Material 1. List of health outcome variables**

| Category | Contents (selected) | Number of items | Example |
| --- | --- | --- | --- |
| Mortality rate | Mortality by cause of death | 530 | Total mortality, Cancer mortality, Diabetes mortality, Hypertension mortality, Infant mortality, Suicide mortality, Number of deaths, etc. |
| Incidence rate | Cancer incidence, Infectious disease, and Injury incidence | 400 | Cancer incidence, Cancer standardized incidence, Number of cancers, Cholera Incidence, Japanese encephalitis incidence,  Hepatitis A incidence, Childbirth complications incidence, etc. |
| Prevalence | Diseases experience rate, obesity rate, oral health | 63 | Atopy prevalence, Hypertension Diagnosis Rate, Diabetes Diagnosis Rate, self-reported obesity rate, Adolescent obesity rate, Dental caries experience, Dental fluorosis, etc. |
| Self-reported health | Self-rated health, Quality of life, activity limitation  Stress, depressive symptoms, suicidal thoughts, cognitive function | 48 | EQ-5D, Self-rated health, Quality of life, Activity limitation, Stress, Depressive symptoms, Suicidal thoughts, Cognitive function, Difficulty Chewing, etc. |
| Others | Traffic accidents, Oral health examination results, violence | 106 | Accident experience rate, Addiction experience rate, Game addiction rate, Falls experience rate, Child abuse rate, Child abuse detection rate, Drunk driving rate, Jaywalking accident rate, child traffic accident rate, etc. |
| Total |  | 1,147 |  |
