# Supplementary material for: Data resource profile: the Korean Community Health Status Indicators (K-CHSI) database
Source: Epidemiol Health. 2023 Feb 2;45:e2023016. doi: 10.4178/epih.e2023016 (PMC10581888; doi:10.4178/epih.e2023016)
Supplement: Supplementary Material 2. — List of health determinants variables [file epih-45-e2023016-Supplementary-2.docx]

**Supplementary Material 2. List of health determinants variables**

| Main category | Subcategory | Number of items | Example |
| --- | --- | --- | --- |
| Sociodemographic factors | Population and household | 76 | Total population, Sex ratio, Population by age group, Daytime population, Population growth rate, Total fertility rate, Crude birth rate, Total number of households, One-person households, Elderly people living alone, etc. |
| Health behavior | Nutrition and diet | 41 | Awareness of Nutrition label, Preference for salty type, Attempting weight loss rate, Subjective obesity awareness rate, etc. |
|  | Alcohol | 30 | Monthly alcohol consumption, Drinking starting age, High Risk Alcohol Consumption, Attempting abstinence rate, etc. |
|  | Health education and public relations | 29 | Smoking prevention education experience rate, Sex education experience rate, High blood pressure management education completion rate, Smoking cessation campaign experience rate, etc. |
|  | Personal hygiene | 14 | Handwashing with Soap, Awareness rate of hand washing to prevent infectious diseases, etc. |
|  | Mental health related behavior | 12 | Mental health counseling rate, Sleep time, Daily time spent online, etc. |
|  | Oral hygiene | 34 | Tooth brushing practice rate, Fluoride toothpaste usage rate, etc. |
|  | Physical activity | 21 | Walking practice rate, Physical activity practice rate, Strength and Resistance Training Exercise practice rate, Sedentary time, etc. |
|  | Safety awareness | 29 | Seat belt wearing rate, Cell phone use while driving, Drunk driving rate, Jaywalking rate, etc. |
|  | Smoking | 46 | Smoking rate, Smoking starting age, Smoking cessation rate, Secondhand smoke exposure rate, Adolescents’ Ease of Cigarette Purchase, etc. |
|  | Screening and vaccination | 145 | Health examination acceptance rate, MMR vaccination rate, Influenza vaccination rate, etc. |
| Social environments | Education | 10 | Number of students per teacher, Number of Kindergarten, Number of universities, etc. |
|  | Economy | 26 | Fiscal independence, Fluctuation rate of land price, Number of businesses, etc. |
|  | Culture | 3 | Culture & Arts Infrastructure, Senior  Welfare Centers, Social welfare facility. |
|  | Welfare | 7 | Government expenditure on social protection,  Percentage of social welfare organizations,  Percentage of public health organizations, Number of child protection agency, etc. |
|  | Social capital | 12 | Mutual trust with neighbors, Religious activity, Social activities, etc. |
|  | Political participation | 3 | Presidential election turnout, Turnout in parliamentary elections, etc. |
|  | Safety | 39 | Local safety level, Number of residents per 119 rescue worker, Number of residents per fire station, etc. |
| Physical environments | Natural environment | 42 | Number of natural disasters, Climate, Wastewater discharge, etc. |
|  | Living environment | 88 | Number of parks, Number of sports facilities, sewerage system, Percent of shared public transport, Number of houses, Vacancy rate, Percentage of old houses, etc. |
|  | Working environment | 19 | Chemical emissions, Cancer-Causing substances emissions, etc. |
| Health care system | Health and medical resources | 155 | Number of medical staff, Number of nursing home, Number of MRI, Number of clinic, Number of AED, etc. |
|  | Health care use | 308 | Unmet health care needs, Number of Emergency Department Use, Health examination acceptance rate, etc. |
|  | Public health service | 34 | Home visiting health service, etc. |
| Total |  | 1,223 |  |
